# Mutation in a putative glycosyltransferase-like gene causes programmed cell death and early leaf senescence in rice

**DOI:** 10.1186/s12284-019-0266-1

**Published:** 2019-02-13

**Authors:** Shanwen Ke, Shuchun Liu, Xin Luan, Xin-Ming Xie, Tzung-Fu Hsieh, Xiang-Qian Zhang

**Affiliations:** 1 Guangdong Engineering Research Center of Grassland Science, College of Forestry and Landscape Architecture, South China Agricultural University, Guangzhou, 510642 China; 2 Plants for Human Health Institute, North Carolina State University, North Carolina Research Campus, Kannapolis, NC 28081 USA; 3 Department of Plant and Microbial Biology, North Carolina State University, Raleigh, NC 27695 USA

**Keywords:** Rice, PCD, Glycosylation, Senescence, Ethylene

## Abstract

Leaf senescence is a genetically regulated, highly complex and ordered process. Although it has been extensively studied, the mechanism of leaf senescence is not well understood. In this study, we isolated a rice mutant, designated as premature senescence leaf (*psl*), which exhibits early senescence and spontaneous lesion mimic phenotype after flowering. The *psl* mutant displays programmed cell death with elevated accumulation of reactive oxygen species (ROS). Molecular and genetic analyses revealed that the phenotypes were caused by a phenylalanine deletion in the *OsPSL* (LOC_Os12g42420) that encode a putative core 2/I branching beta-1,6-N-acetylglucosaminyl transferase predicted to be involved in protein glycosylation modification. *OsPSL* mRNA levels increased as senescence progressed, with maximum accumulation of transcripts at late senescence stages in WT plants. Moreover, remarkedly down-regulated transcriptional levels of O-linked N-acetylglucosamine (O-GlcNAc) transferases (OGTs) genes were observed in *psl* mutant, supporting the occurrence of impaired O-glycosylation modification. Proteomic analysis showed that ethylene-related metabolic enzymes including S-adenosyl methionine (SAM) synthetase (SAMS) were significantly upregulated in the *psl* mutant compared with WT. Consistent with the proteomic results, ethylene concentration is higher in *psl* mutant than in wild-type plants, and transcript levels of ethylene synthesis and signal transduction genes were induced in *psl* mutant. The early leaf senescence of *psl* can be partially rescued by ethylene biosynthesis inhibitor aminoethoxyvinylglycine treatment. These results highlight the importance of protein O-glycosylation in PCD and leaf senescence, and suggest a possible role of OsPSL in ethylene signaling.

**Electronic supplementary material:**

The online version of this article (10.1186/s12284-019-0266-1) contains supplementary material, which is available to authorized users.

## Background

Leaf senescence is a highly complex and programmed process and involves a series of cytological and biochemical changes. The onset of leaf senescence depends on developmental programs and environmental signals; i.e., it occurs in an age-dependent manner. Consequently, young leaves are insensitive to senescence-inducing signals (Jibran et al., 2013). In annual plants, flowering is associated with senescence and death of the whole plant. During senescence, photosynthetic pigments such as chlorophyll and carotenoids are degraded and thus yellowing of leaves is a morphological indicator of senescence (Thakur et al., 2016). On the other hand, leaf senescence can also be accelerated due to various environmental stresses including pathogen attack. In plants, one of the most common defense responses to pathogen attack is the hypersensitive response (HR). It has been shown that HR results from programmed cell death (PCD) process, as evidenced by the existence of mutants that spontaneously activate the HR in the absence of a pathogen (Dangl et al., 1996). The lesion mimic mutants (LMM) exhibiting HR lesions in the absence of pathogens are thought to be defective in genes that control PCD, representing a powerful tool for the study of PCD in plants. Besides induced by pathogen, PCD is also essential for growth and development in animals and plants, including leaf senescence (Trobacher, 2009). However, the molecular mechanisms of PCD and leaf senescence are still not well understood in plants.

More than 50% of the proteins in eukaryotes are glycoproteins (Gomord et al., 2010). Protein glycosylation can be classified into two main categories: *N*-linked glycosylation at the amide group of asparagine (Asn) residues and *O*-linked glycosylation at the hydroxyl group of serine (Ser), threonine (Thr), hydroxylysine or hydroxyproline (Hyp) residues in the protein chain (Gomord et al., 2010). There are multiple types of protein O-glycosylation. Mucin-type O-glycans is the most abundant O-glycans in humans. Mucin-type O-glycosylations can be further classified into eight major groups (core1-core8) (Moran et al., 2011; Ye et al., 2015). In general, O-linked glycans contain 1–20 carbohydrate residues, which are shorter than N-linked glycan chains. For core1-core4 mucin-type O-glycans, the carbohydrate chain is initiated with an N-acetylgalactosamine (GalNAc) residue linked to Ser or Thr. For example, core2 mucin-type of O-glycosylation starts with the addition of a GalNAc residue onto the hydroxyl groups of Ser or Thr. Core 2 β-1,6-N-acetylglucosaminyltransferase-1 (C2GNT) catalyzes the transfer of N-acetylglucosamine from uridine diphosphate-N-acetylglucosamine with a β1,6-linkage to α-N-acetylgalactosamine of a core 1 O-glycan (Kojima et al., 2015). For core5-core8 mucin-type O-glycans, contain one residue. For example, *O*-GlcNAcylated proteins consist of a single *O*-linked N-acetylglucosamine on serine and threonine residues belong to core8 mucin-type glycosylated protein (Xu et al., 2017).

Several studies in *Arabidopsis* have uncovered functions for O-glycosylation in multiple important biological processes during plant development including flowering and epigenetic modification (Zentella et al., 2016; Xu et al., 2017; Zentella et al., 2017; Xing et al., 2018). In this paper, we characterized an early senescence rice mutant *psl* with HR-like lesions in the absence of pathogen attacks. Using a map-based cloning approach, we determined that *OsPSL* encodes a putative member of the core 2/I branching beta-1,6-N-acetylglucosaminyl transferases family, which is involved in protein *O*-glycosylation modification. Our study provides an important evidence for a key role of a putative acetylglucosaminyltransferase in regulating leaf senescence in rice.

## Methods

### Plant materials

A spontaneously occurring rice early senescence mutant *psl* was isolated from the *Oryza sativa* L. ssp. *Japonica* cultivar Zhonghua 11.

### Histochemical staining and quantification of ROS

Histochemical staining was performed on fresh leaves as previously described (Qiao et al., 2009) with modifications. In brief, fresh leaf examples were vacuum infiltrated in 0.5 mg ml^− 1^ nitro blue tetrazolium (NBT) or 1 mg ml^− 1^ 3,3^′^-diaminobenzidine (DAB) for 10 min, and then left at room temperature for 12 h in the dark. After staining, the chlorophyll was extracted by soaking the samples in 90% ethanol for 3 h at 42 °C or until the green pigment was completely removed. H_2_O_2_ and O_2_^−^ levels in the flag leaves two days after flowering were quantified according to Yang et al. (Yang et al., 2016).

### DNA laddering

Flag leaves from wild-type and *psl* plants were collected at three different stages: 10 days before flowering, and 2 or 7 days after flowering. The DNA extraction was conducted using a convenient method as previously described (Zhang et al., 2013) with modifications. In brief, a small piece of leaf tissue ground to a fine powder (approximately 100 mg) was incubated with 1000 μL of buffer at 75 °C for 30 min. Following centrifugation at 12,000 rpm for 10 min, 500 μL of the supernatant was transferred to fresh tubes and the DNA was precipitated with 500 μL of islpropanol with 50 μL sodium acetate buffer (pH 5.2). For the DNA fragmentation assay, ~ 10 μg of genomic DNA was separated by electrophoresis on a 1.5% agarose. Extraction buffer: 100 mM Tris-HCl at pH 8.0, 10 mM EDTA at pH 8.0, 1 M KCl.

### Map-based cloning

For map-based cloning of the *OsPSL* gene, 692 individual plants showing early leaf senescence were selected from an F_2_ population derived from a cross between the *psl* mutant and *indica* var. Huajingxian74. Bulk segregant analysis (BSA) was first performed for preliminary genetic mapping (Michelmore et al., 1991). For BSA, two DNA bulks were constructed by mixing an equal amount genomic DNA from 20 wild-type and mutant plants from the F_2_ mapping population, respectively. Simple sequence repeats (SSRs) were identified using SSRHunter software (Li and Wan, 2005). For fine mapping, insertion-deletion (InDel) markers were obtained according to the rice DNA polymorphic database between Nipponbare (*O. sativa* ssp. *japonica*) and 93–11 (*O. sativa* ssp. *indica*) (Shen et al., 2004). Genomic DNA was extracted from young leaves of each parent and F_2_ individuals using a convenient method (Zhang et al., 2013). The *OsPSL* gene was selected from an approximately 47.8-kb region as the candidate gene. To find out the mutation site, we amplified the corresponding fragments from the *psl* mutant and wild-type plants, respectively. Primers used for the map-based cloning were listed in Additional file 1: Table S1.

### Vector constructs and rice transformation

For complementation of the *psl* mutation, the full-length coding sequence of *OsPSL* was amplified using the following primers: PLSF (GGTACCTCTAGAATGGCGCTGCCGCACGCCGCCT) and PLSR (ACTAGTCTACCATGGGTCTCGCAGGATGACGGA). The 1098-bp PCR product was cloned into pCUbi1390 binary vector. The resulting plasmid was introduced into the calli generated from the mature seed embryos of the *psl* mutants through the *Agrobacterium tumefaciens* (strain EHA105)-mediated method (Hiei et al., 1994).

### Gene expression analysis

Total RNA was extracted from frozen samples using TRIzol reagent (Invitrogen) according to the manufacturer’s instructions. After RNase-free DNase treatment, and first strand cDNA was generated using a Revert Aid First Strand cDNA Synthesis Kit (Thermo Scientific). Quantitative RT-PCR (qRT-PCR) was performed using a SYBR Premix Ex Taq™ RT-PCR kit (Takara) following the manufacturer’s instruction. The primers for qRT-PCR are shown in Additional file 1: Table S1. The rice *25sRNA* gene was used as an internal control for all analysis. Three replicates were performed for each analysis, and average values and standard deviations are shown.

### Proteome analysis

Identification of differentially expressed proteins was performed by Fitgene Biotech CO., Ltd. (Guangzhou, China) using two-dimensional gel electrophoresis (2-DGE) as previously described (Kim et al., 2008; Chen et al., 2013a) with some modifications. In brief, the samples of flag leaf two days before flowering were collected and total proteins were extracted from collected leaf samples of 10 plants with 3 bioreplicates. The 2-D gels were stained with Coomassie Blue G250 (Bio-Rad). Gels were scanned (300 dpi, 16-bit grayscale pixel depth, TIFF file) for image/data analysis performed using the Image Master 2D Platinum imaging software ver. 5.0. After quantitative detection, the significant differences of (> 2 folds, *p* < 0.05) protein spots were marked and selected for further identification by MS if they were confirmed in three independent sample sets.

Mass spectra were acquired on a MALDI-TOF/TOF mass spectrometer, the Bruker-Daltonics AutoFlex speed™ MALDI-TOF/TOF (Bruker, Germany). Peptide mass values were compared using the software flexAnalysis (Bruker Dalton). Protein database searching was performed with the MASCOT search engine against the NCBI nonredundant protein database (http://www.ncbi.nlm.nih.gov/). The species selected was *Oryza sativa*.

### Quantification of ethylene and AVG treatment

Leaf samples were collected 0, 3, 5, 7 and 10 days after flowering, and three leaves were placed into a 15 mL glass vial sealed with a gas-proof septum. After imbibition in a growth cabinet at 26 °C for 30 h, a 0.1 mL gas sample was withdrawn from the head space of each bottle using a gas-tight syringe (Hamilton), and the ethylene concentration was determination by gas chromatography (Shimaduz GZ-14CPF). The quantified data, divided by fresh weight and time, were converted to specific activities.

For phenotype analysis of the leaves in response to the exogenous ethylene inhibitor, wild-type and *psl* plants were sprayed 5 day before flowering with 50 μM aminoethoxyvinylglycine (AVG), an inhibitor of ethylene biosynthesis, for 20 days (once per day) in a paddy field.

## Results

### Phenotypic characterization of rice *psl* mutant

**Fig. 1:**
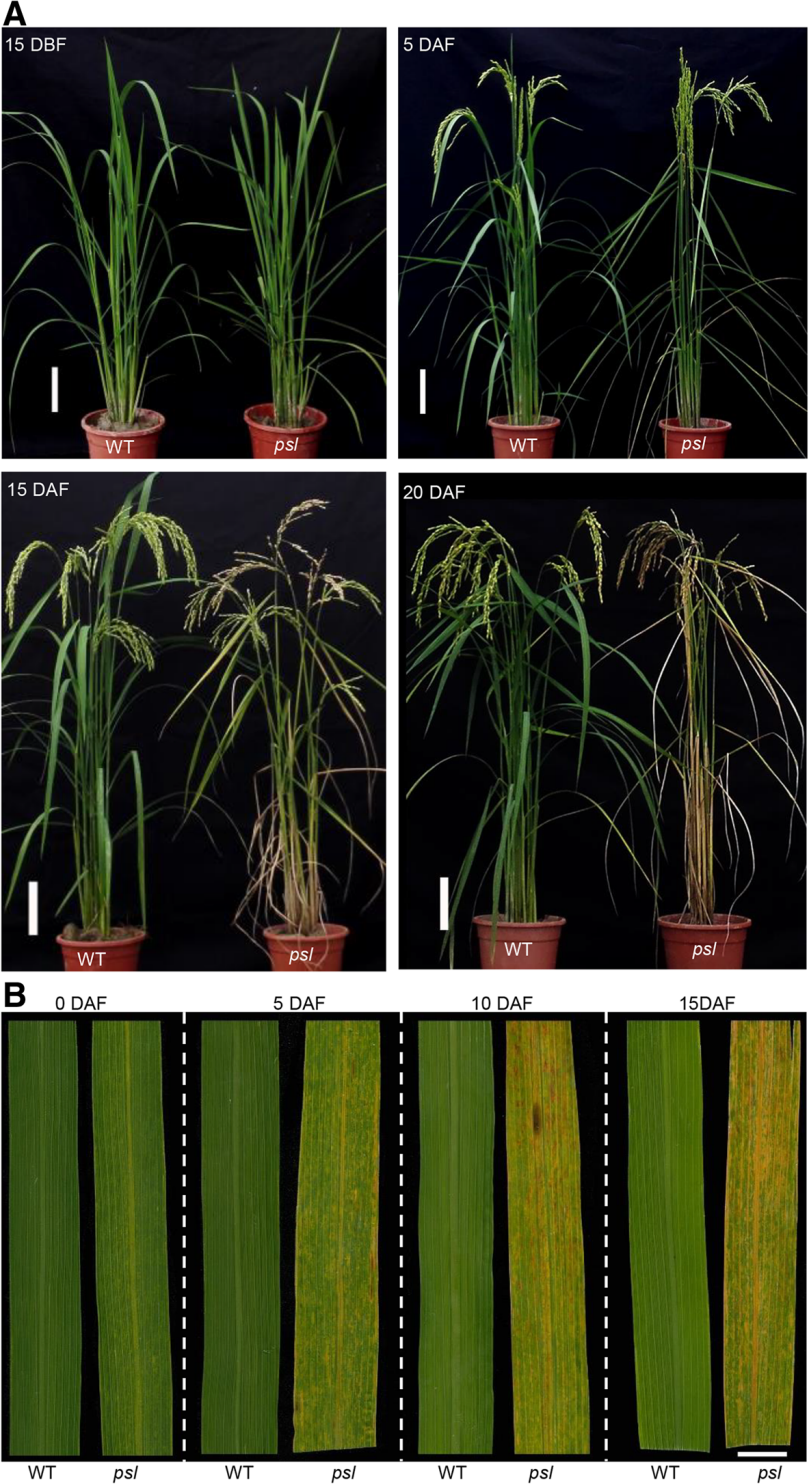
Phenotype of the *psl* mutant. **a** Comparison of leaf phenotype in wild-type and *psl* mutant plants at different developmental stage. **b** Comparison of lesion mimic phenotype in wild-type leaves and *psl* mutant leaves. DBF: days before flowering; DAF: days after flowering. Bar = 10 cm in (A) and 1 cm in (B)

A naturally occurring rice mutant with an early leaf senescence phenotype, named premature senescence leaf (*psl*), was isolated from a *japonica* variety Zhonghua 11. Under paddy field conditions, the *psl* plants were almost indistinguishable from wild type (WT) plants before heading (Fig. 1a). After flowering, the *psl* mutant plants exhibited accelerated senescence compared with wild-type plants. Leaves displayed chlorosis at the early flowering stage, began to wilt at approximately 15 days after flowering, and eventually died at three weeks after flowering (Fig. 1a). Unlike the yellowing of leaves due to naturally senescence, small yellow lesions appeared on the leaves of *psl* mutant plants, whereas the leaves of wild-type plants at the same stage exhibited no such abnormal symptoms (Fig. 1b). In addition, the *psl* mutants had almost the same plant height and heading date but had obviously reduced seed setting rate and 1000-grain weight (Additional file 1: Table S2).

To determine the inheritance of *psl*, we examined the phenotypes of progeny derived from a cross between *psl* and wild type (Zhonghua11). All F_1_ plant displayed wild-type phenotype, and their F_2_ progenies showed a segregation ratio of 3:1 (normal: early leaf senescence = 442:130; χ2 = 1.46 < χ2_0.05_ = 3.84), indicating that the mutant phenotype was controlled by a single recessive nuclear locus.

### Accumulation of reactive oxygen species (ROS) in *pls*

**Fig. 2:**
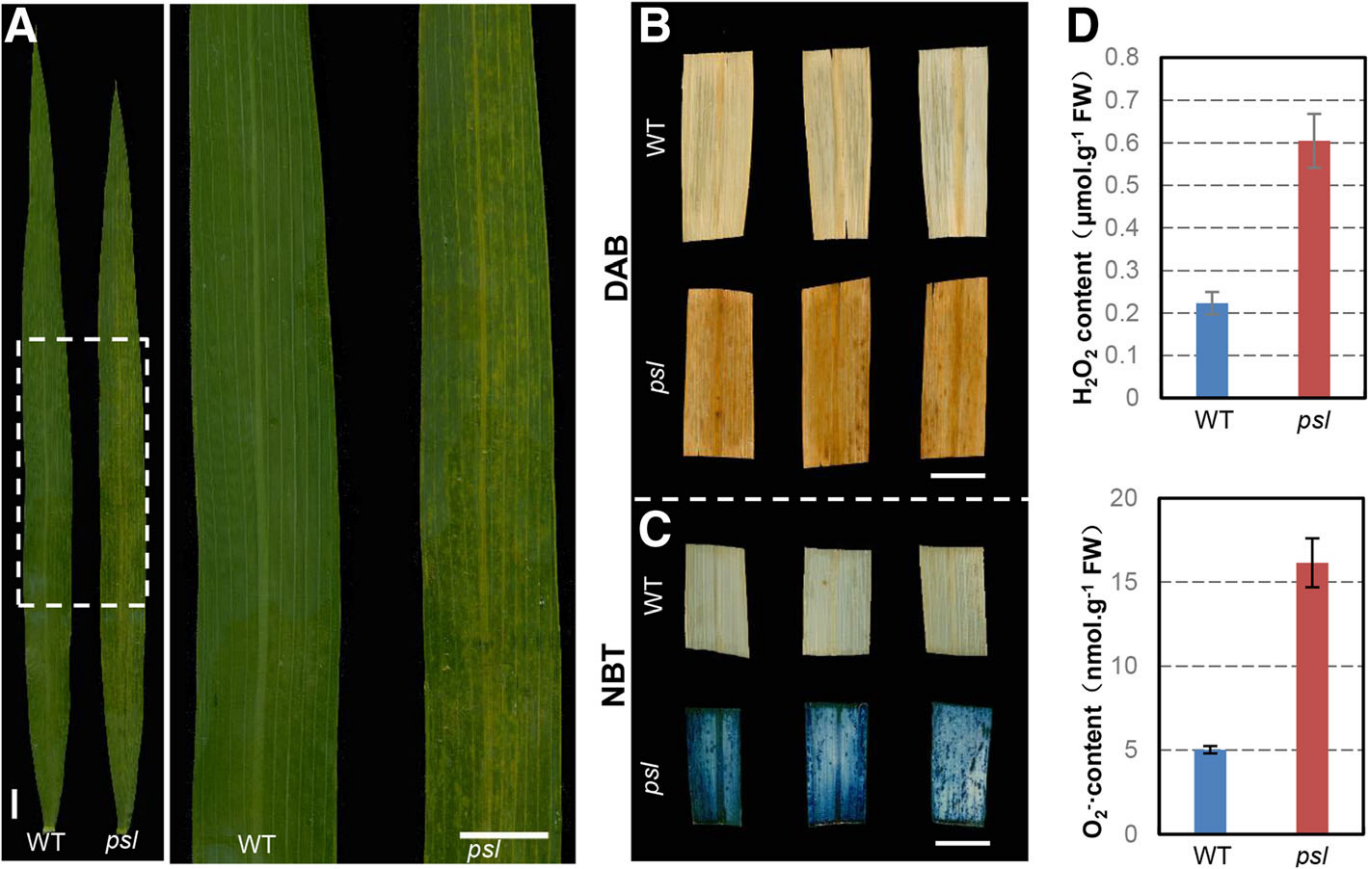
Comparison of ROS levels between wild-type and *psl* plants. **a** phenotype of flag leaves two days after flowering. **b** Leaves stained with 3,3′-diaminobenzidine (DAB), showing the H_2_O_2_ levels in wild-type (WT) and *psl* plants*.* **c** Leaves stained with nitroblue tetrazolium (NBT), showing O_2_^−^ in WT and *psl* plants. **d** ROS (H_2_O_2_ and O_2_^−^) content in *psl* mutant. Scale bar, 1 cm

To determine possible biochemical mechanisms involved in the early senescence and HR-like lesions in the *psl* mutant, we compared the ROS accumulation of flag leaves two days after flowering (H_2_O_2_ and O_2_^−^) between *psl* and wild-type plants by 3,3′-diaminobenzidine (DAB) and nitroblue tetrazolium (NBT) staining. As shown in Fig. 2, both H_2_O_2_ and O_2_^−^ are significantly increased in *psl* leaves, suggesting that the accumulation of ROS may play an important role in triggering cell death in *psl*.

### DNA fragmentation analysis in mutant plants

**Fig. 3:**
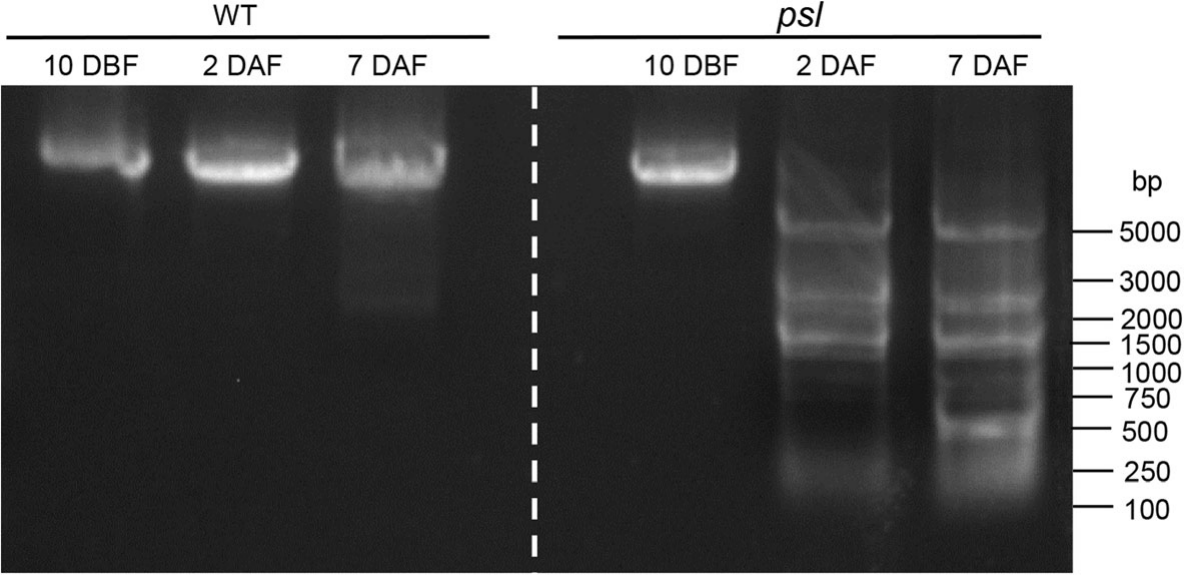
DNA fragmentation in *psl* mutant. The DNA ladder of *psl* plants was detected by DNA gel blot analysis. Equal amounts of total DNA isolated from *psl* and wild-type plants at the same developmental stage were loaded in each lane. Positions of DNA standards (identified in bases) are indicated at right. 10 DBF: 10 days before flowering; 2, 7 DAF: 2 and 7 days after flowering

DNA fragmentation is a hallmark of PCD (Bröker et al., 2005). To determine whether the mutation of *OsPSL* cause PCD in *psl* mutant, we conducted the total DNA analysis of flag leaf of WT and *psl* plants at different stages (10 days before flowering, 2 and 7 days after flowering). DNA gel blot analysis showed an apparent DNA ladder in the total DNA isolated from *psl* plants after flowering, whereas this pattern of DNA fragmentation was barely detectable in wild-type plants at an identical developmental stage (Fig. 3).

### *OsPSL* encodes a putative acetylglucosaminyl transferases

**Fig. 4:**
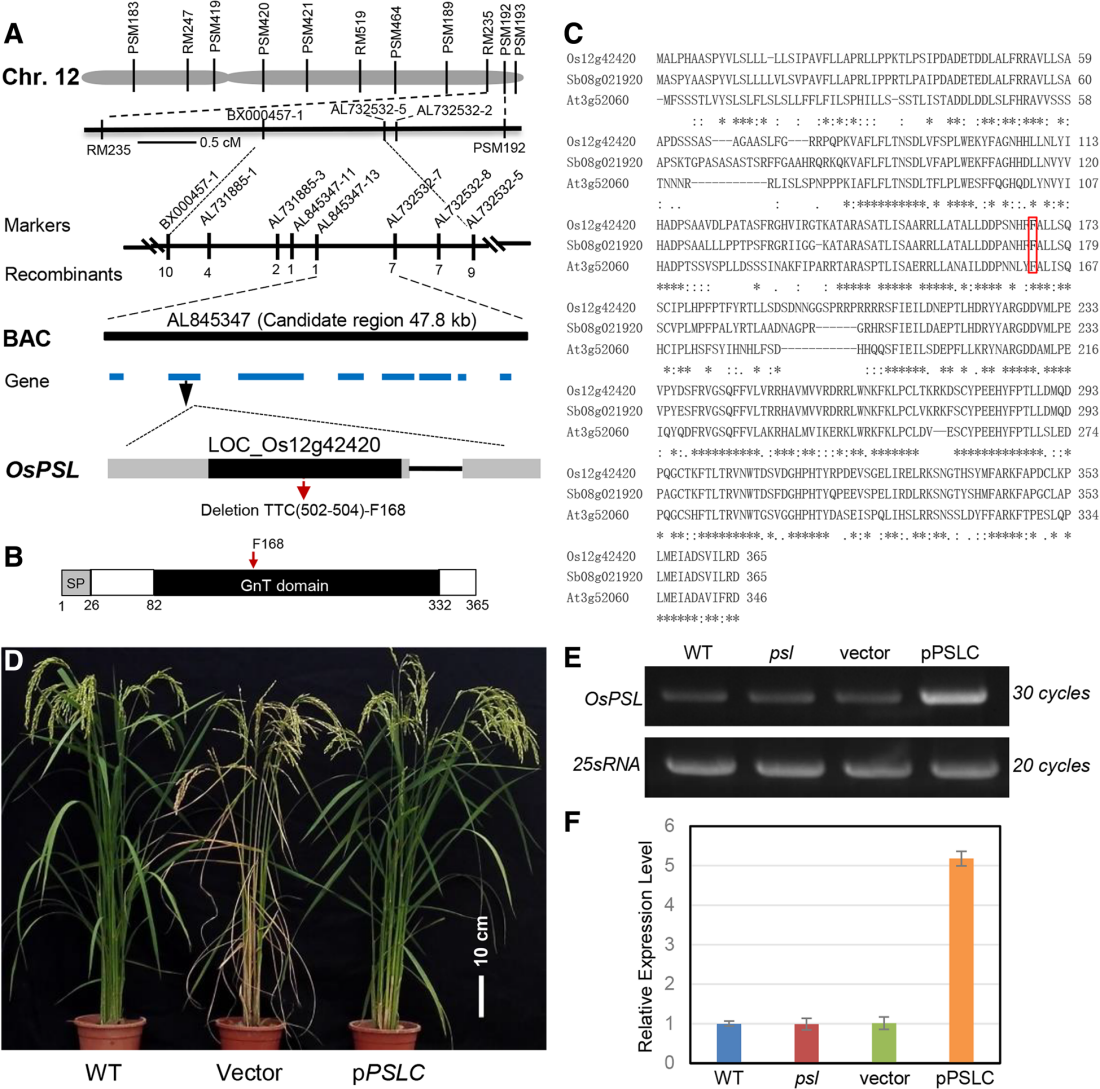
Map-based cloning of the *OsPSL* locus. **a** The *PSL* locus was mapped to the long arm of rice chromosome 12. Black boxes indicate the coding sequence, grey boxes indicate the 5′ and 3′untranslated regions, and lines between boxes indicate introns. Deletion site identified in the *psl* are indicated by a red arrowhead. **b** Schematic representation of the OsPSL protein domains. Positions of amino acid residues delimiting each of the indicated domains are shown. **c** Amino acid sequence alignment of the rice OsPSL (Os12g42420) with its homologs from *Arabidopsis*. *thaliana* (At3g52060) and *Sorghum. bicolor* (sb08g021920). Symbol designations: “*” identical residues, “:” conserved substitutions, “.” semi-conserved substitutions. The conserved phenylalanine residue likely involved in the enzymatic activity is indicated by a red box, and gaps introduced for alignment are indicated by dashes. **d** Complementation analysis of the *psl* mutant. Plant phenotype of WT and *psl* mutant transformed with *OsPSL* (pPSLC) or with empty vector (Vector) grown in a paddy field. Expression analysis of *OsPSL* gene in WT, *psl* mutant and the transgenic plants with *OsPSL* (pPSLC) or with empty vector (Vector) by semi-quantitative (**e**) and quantitative (**f**) RT-PCR

To identify the mutated gene responsible for the *psl* phenotypes, map-based cloning method was employed. Initially, BSA was conducted with 220 SSR markers distributing with 5–10 cM intervals on 12 chromosomes. BSA revealed that *OsPSL* was located on the long arm of chromosome 12, and one SSR marker, PSM193, was closely linked to the *OsPSL* locus. Subsequently, linkage analysis was conducted by genotyping 870 F_2_ populations with several additional SSR markers located on chromosome 12, and the location of the *OsPSL* locus was identified inside two makers (RM235 and PSM192) on chromosome 12, with genetic distances of 1.2 cM and 0.5 cM, respectively. For fine mapping of *OsPSL*, > 3000 F_2_ mapping populations consisting of 692 mutant individuals were developed from the cross between Huajingxian74 and the *psl* mutant. InDel markers were designed according to sequence differences between *indica* and *japonica* rice. Ultimately, the *OsPSL* locus was mapped to a 47.8 kb DNA region on chromosome 12 between InDel marker AL845347–13 and AL732532–7 (Fig. 4a). Within this 47.8-kb interval, there are eight predicted ORFs, including LOC_Os12g42420. DNA sequence comparison revealed a 3-bp deletion in the first exon of LOC_Os12g42420 in the *psl* and no sequence difference was found in the seven other genes (Additional file 1: Figure S1). LOC_Os12g42420 contains 1098 nucleotides of CDS and encodes predicted polypeptides of 365 amino acid residues with a calculated molecular mass of 41.03 kD. The 3-bp deletion in the *psl* mutant occurred at 502–504 bp downstream from the initiation codon ATG and is predicted to encode a phenylalanine (F168) residue (Fig. 4a).

Amino acid sequence analysis revealed that OsPSL (LOC_Os12g42420) belongs to the annotated family of core 2/I branching beta-1,6-N-acetylglucosaminyl transferases (Yang et al., 2017). OsPSL contains two functional domains, an amino-terminal signal peptide and a catalytic domain (GnT) of a Branch family/glycosyltransferase family 14 (Fig. 4b). Further analysis demonstrated that OsPSL contains a conserved phenylalanine residue (F168 in OsPSL) potentially important for the enzymatic activity (Fig. 4c). Three-dimensional structure prediction by using Phyre2 server (http://www.sbg.bio.ic.ac.uk/phyre2) revealed that the F168 residue was located in a *β*-sheet and its deletion led to the absence of the *β*-sheet in OsPSL protein (Additional file 1: Figure S2).

To confirm the mutation in LOC_Os12g42420 was responsible for the abnormal phenotypes of *psl* mutants, we performed a genetic complementation test. An overexpression vector (pPSLC) containing the entire open reading frame (ORF) of *OsPSL* was constructed and introduced into *psl* mutants by *A. tumefaciens*-mediated transformation (Fig. 4d). The empty vector pCUbi1390 was also introduced to *psl* mutants as a control. We found that the *psl* mutant phenotype was rescued in 18 independent pPSLC transgenic plants with the overexpression of wild-type *OsPSL* compared to WT and *psl* mutant, while the empty vector failed to complement the *psl* mutant phenotype in 15 lines (Fig. 4d-f). The findings confirmed that mutation in LOC_Os12g42420 is responsible for the mutant phenotype.

### Expression pattern *of OsPSL*

**Fig. 5:**
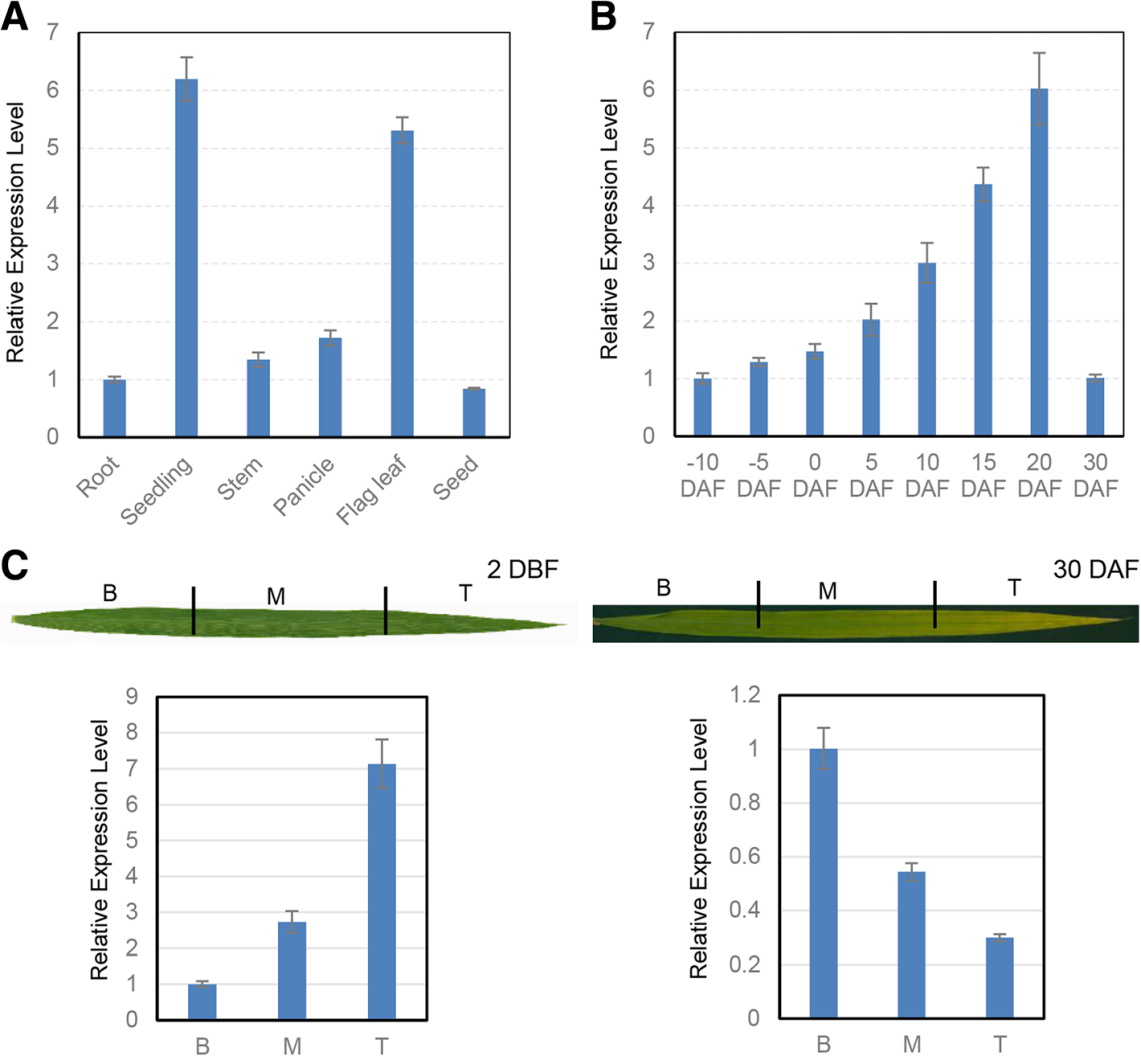
Analysis of *OsPSL* expression. **a** Quantitative RT-PCR (qRT-PCR) analysis of *OsPSL* expression in various organs, including root and young seedling at the two-leaf stage, stem, panicle and flag leaf at booting stage, and seed 10 days after flowering (DAF). **b** Change over time in the *OsPSL* transcription levels of flag leaves. **c** *OsPAL* expression in different parts of the fully expanded flag leaf 2 days before flowering (DBF) and 30 DAF. B, base; M, middle; T, tip

Expression analysis showed that *OsPSL* was expressed constitutively across tissue types, but with preferential expression in seedling and leaf blade (Fig. 5a). Further we examined *OsPSL* expression in the leaves at different developmental stages. A kinetic analysis of *OsPSL* expression in flag leaves showed that, although the number of *OsPSL* transcripts increased gradually before heading, it increased dramatically just after flowering, while decreased rapidly in old leaf 30 days after flowering (Fig. 5b). Rice leaf matures from the tip to the base, displaying a developmental gradient, is an attractive model system for developmental studies. Consistent with this observation of different developmental stages, *OsPSL* expression increased gradually from the base to the tip of a fully expanded flag leaf two days before flowering, but decreased gradually from the tip to the base in the flag leaf 30 days after flowering, displaying yellowing of leave as result of the degradation of photosynthetic pigments (Fig. 5c). The results suggest that *OsPSL* plays an important role in delaying leaf senescence.

### Expression of O-glycosylation related-genes is altered in the *psl* mutant

**Fig. 6:**
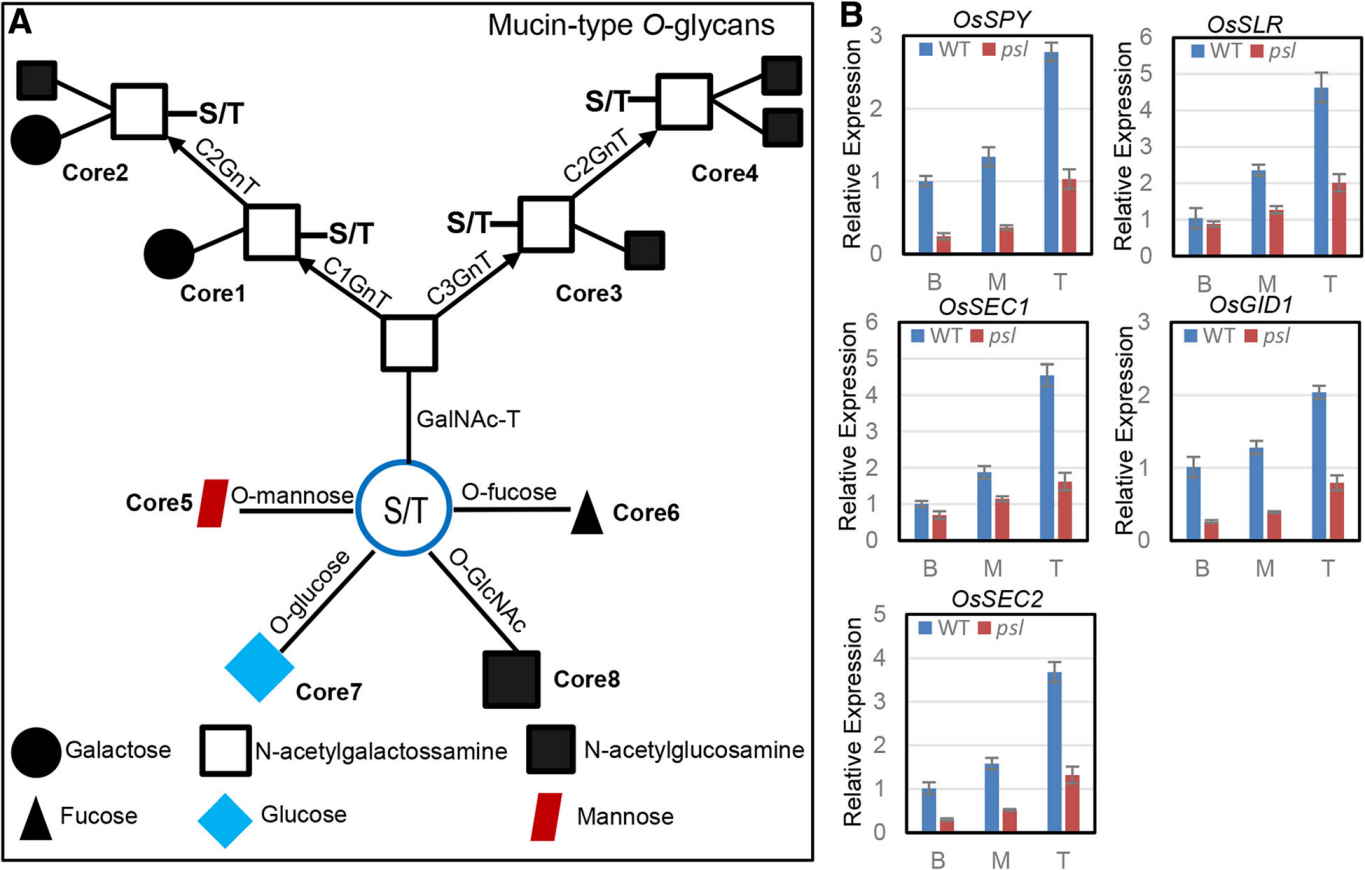
Mutation of *OsPSL* has an effect on the expression of rice putative O-GlcNAc transferases (OGTs) genes. **a** Illustration of mucin-type O-glycan synthesis. **b** Expression analysis of *O*-linked glycosylation-related genes in different parts of the flag leaf in WT and *psl* mutant two days before flowering. B, base of leaf; M, middle of leaf; T, tip of leaf

Mucin-type O-glycosylations are divided into eight major groups based on different carbohydrate residues (Moran et al., 2011; Ye et al., 2015) (Fig. 6a). Mucin-type core 8 *O*-glycn is biosynthesized by *O*-linked *N*-acetylglucosamine (O-GlcNAc) transferase (OGT). *O*-linked *N*-acetylglucosamine (O-GlcNAc) modification affects diverse plant processes including response to hormones and environmental signals. Plants have two putative distinct O-GlcNAc transferases (OGTs), SEC- and SPY-like (Olszewski et al., 2010). Recently, it has been demonstrated that AtSEC acts as O-GlcNAc transferase whereas AtSPY is a protein O-fucosyltransferase in *Arabidopsis* (Zentella et al., 2016; Zentella et al., 2017). Rice has three putative OGTs genes, one SPY-like and two SEC-like genes (Shimada et al., 2006). To determine whether the mutation of *OsPSL* has an effect on the expression of rice OGTs, we conducted the transcript analysis of rice OGTs. As shown in Fig. 6b, three of rice OGTs genes, *SPY*, *SEC1* and *SEC2*, were significantly downregulated compared to the wild-type plants. Given that the *Arabidopsis* DELLA protein RGA, a direct target of OGTs (Zentella et al., 2017), is a repressor in GA pathway, we examine the expression of rice *SLR* and *GID1* genes, encoding rice DELLA protein and GA receptor, respectively, in wild-type and *psl* plants. The abundances of the transcripts of *SLR* and *GID1* were also reduced in the *psl* mutant. The findings suggest that proteins glycosylation modification might be altered in *psl* mutant, consequently influencing GA signaling.

### Proteome analysis of the rice *psl*

**Fig. 7:**
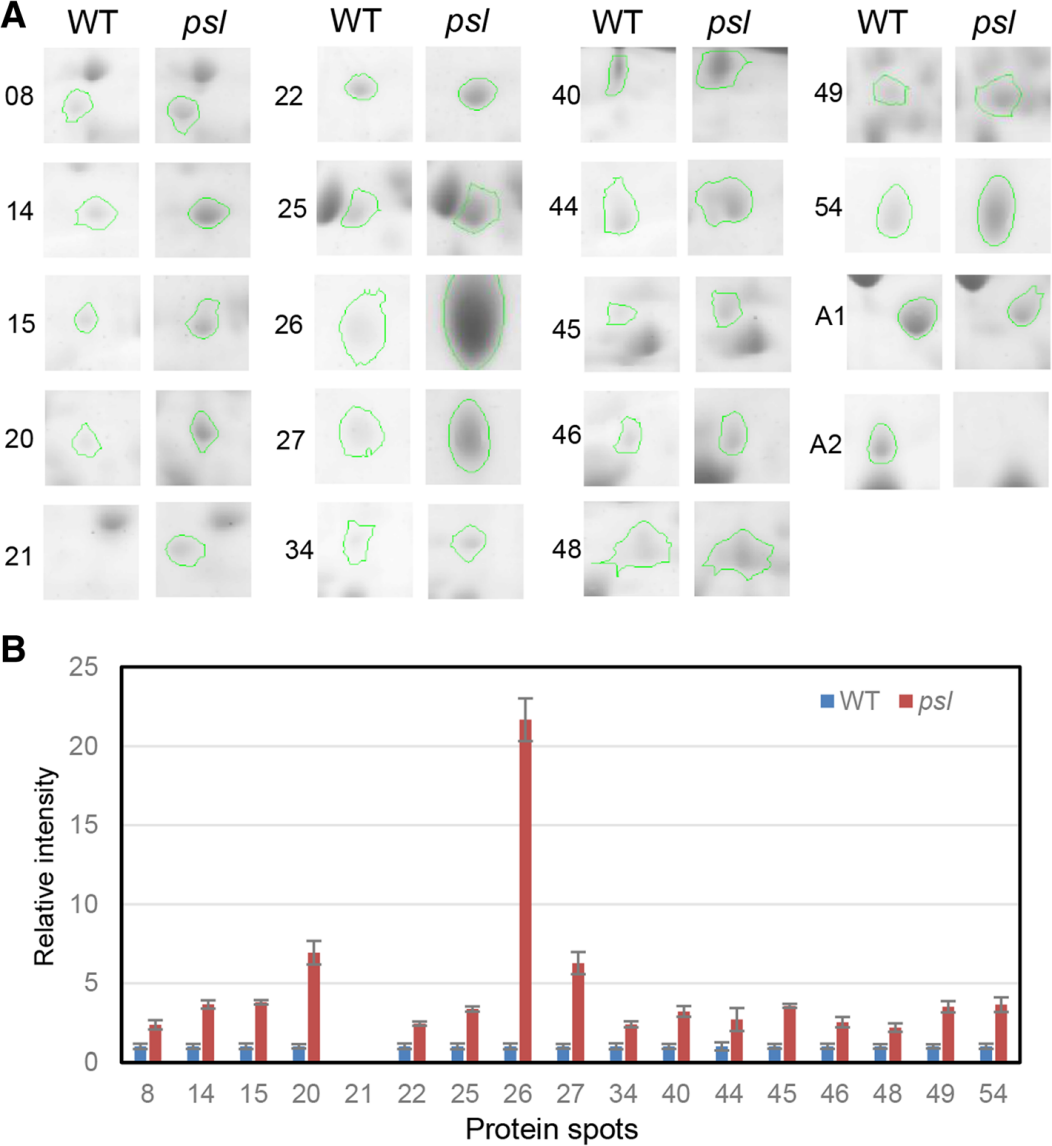
2-DE analysis between WT and *psl* mutant. **a** Magnified regions of 2-DE gels. Numbers at the left of images indicate protein spots showed by arrows in Additional file 1: Figure S1 with significantly differential accumulation levels between *psl* and WT mutant in 2-DE gel analysis. **b** Quantitative analysis of differentially induced proteins on the 2-D gels. The mean relative expression level of three replicate samples is shown in the histograms based on relative protein intensities compared with background levels. Quantification of 19 protein spots from samples was made with ImageMaster 2D Platinum. Error bars indicate the standard deviation

To understand the molecular mechanisms of *PSL* mutation-induced cell death, we compared the protein profiles of *psl* mutant and WT by two-dimensional gel electrophoresis (2-DE) (Additional file 1: Figure S3) and found that 19 proteins were differentially accumulated (> 2-fold changes) between WT and *psl* mutant two days before flowering (Fig. 7). Among these 19 proteins, 17 were up-regulated and 2 were down-regulated, respectively, in *psl* mutant, which could be categorized into different functional classes (Table 1). Among them, some proteins including photosynthesis, metabolic enzymes, defense and stress-related proteins are also differentially expressed in LMMs. For example, probenazole-induced protein (PBZ1)/pathogen-related protein 10 (OsPR10, spot 26), which is a molecular marker in rice defense and stress response and can serve as a potential marker for cell death/PCD in rice (Kim et al., 2008), were highly induced in the *psl* mutant (Fig. 7). In addition, other defense/stress-related proteins such as thaumatin-like protein/OsPR5 (spot 27) were also remarkedly induced in the *psl* mutant, as observed in lesion mimic mutant *spl1*(Kim et al., 2008) and *blm* (Jung et al., 2006) .

**Table 1:** Identification of proteins differentially expressed between WT and *psl* mutant

Function type	Spot ID	Homologous protein	Score	Coverage (%)	pI	MM (kDa)	Chang fold	ID
Photosynthesis	A01	Rubisco activase alpha form (Rubisco-A)	394	50	5.36	38.69	−2.57	LOC_Os11g47970
ROS metabolism	A02	glutathione S-transferase OsGSTF3	143	38	5.81	25.51	≪	LOC_Os03g04260
ROS metabolism	B25	glutathione S-transferase	315	35	5.50	25.34	3.36	LOC_Os09g29200
ROS metabolism	B44	GDP-d-mannose 3″,5″-epimerase (GME)	262	35	5.75	43.15	2.70	LOC_Os10g28200
ROS metabolism	B45	Monodehydroascorbate reductase (MDAR)	253	31	5.30	46.77	3.56	LOC_Os08g44340
Amino-acid metabolism	B08	Methylenetetrahydrofolate reductase (MTHFR)	260	32	5.75	64.66	2.36	LOC_Os03g60090
Amino-acid metabolism	B14	S-adenosylmethionine synthase (SAMS1)	292	34	5.83	43.00	3.65	LOC_Os05g04510
Amino-acid metabolism	B15	S-adenosylmethionine synthase (SAMS2)	355	35	5.68	43.33	3.79	LOC_Os01g22010
Amino-acid metabolism/	B46	Aspartate aminotransferase (AST)	411	46	5.90	46.02	2.53	LOC_Os02g55420
GA-related	B20	gibberellin receptor GID1L2	360	49	5.26	36.24	6.93	LOC_Os07g06830
Ca^2+^ signal	B21	Calcium-dependent lipid-binding (CaLB) protein	308	46	5.97	30.51	≫	LOC_Os09g39770
Ca^2+^ signal	B22	Calcium-dependent lipid-binding (CaLB) protein	309	42	4.77	30.38	2.44	LOC_Os08g44850
Defense-related	B26	OsPR10a/PBZ1	202	46	4.88	16.91	21.68	LOC_Os12g36880
Defense-related	B27	thaumatin-like protein (OsPR5)	190	22	5.07	18.55	6.27	LOC_Os12g43380
Stress-related	B34	PDI-like protein/nucleoredoxin	231	28	4.95	64.05	2.39	LOC_Os03g29190
Stress-related	B54	Translationally-controlled tumor protein (TCTP)	283	55	4.51	18.99	3.64	LOC_Os11g43900
pentose phosphate pathway	B40	6-phosphogluconate dehydrogenase (Os6PGDH)	215	35	5.85	52.97	3.21	LOC_Os06g02144
Glycolysis	B49	Glyceraldehyde-3-phosphate dehydrogenase (GAPDH)	159	19	6.22	47.54	3.51	LOC_Os03g03720
Chloroplast protein	B48	Import intermediate associated protein IAP100	94	21	6.57	40.45	2.19	LOC_Os10g35030

≪, much less than; ≫, much greater than

Recent studies reveal that Glyceraldehyde-3-phosphate dehydrogenase (GAPDH) also mediates cell death by its nuclear translocation under oxidative stress in mice (Nakajima et al., 2009). Increased level of GAPDH protein was also observed in lesion mimic mutants (lmms) *spl1*, *spl5* and *cdr2* (Chen et al., 2013b), suggesting its function in cell death and defense responses in rice. Besides metabolic function, 6-phosphogluconate dehydrogenase (Os6PGdh) plays a role in abiotic stresses in rice (Hou et al., 2007). Two enzymes of glycolysis, GAPDH and Os6PGDH, were up-regulated in *psl* mutant (Table 1), supporting the notion that the key enzyme of glycolysis metabolism may be involved in cell death and defense response in rice.

In differently expressed proteins, four enzymes including Methylenetetrahydrofolate reductase (MTHFR; up), aspartate aminotransferase (AST; up) and S-adenosyl-L-methionine (SAM) synthetase (SAMS1 and SAMS2; up) are associated with SAM metabolism (Table 1). MTHFR reduces 5,10-methylenetetrahydrofolate to 5-methyltetrahydrofolate, which is used to convert homocysteine to methionine (Goyette et al., 1994). Aspartate is converted to homocysteine by a series of enzymes including AST and is also linked to methionine metabolism (Hesse and Hoefgen, 2003). SAM synthase (SAMS) plays the role of catalyzing the synthesis of SAM from methionine and ATP (Chu et al., 2013). Because SAM acts as the precursor in the biosynthesis of the plant hormone ethylene (Roje, 2006), the findings imply that the mutation of *OsPSL* might alter SAM metabolism, in turn affecting ethylene biosynthesis.

The induction of many defense-related proteins, such as PBZ1/OsPR10, thaumatin-like protein/OsPR5 and translationally controlled tumor protein (TCTP), was also found in the leaves of the *psl* mutant. Some proteins are not only stress-related/PR proteins, but also involved in ethylene response. For example, in tobacco (*Nicotiana tabacum*), NtTCTP protein accumulation is induced by ethylene treatment and interacts with a subfamily II ethylene receptor Histidine Kinase1 (NTHK1) to regulate plant growth and response to ethylene (Tao et al., 2015). In rice, TCTP is a single-copy gene that has diverse roles in response to various stresses (Wang et al., 2015). Thus, the induction of TCTP in *psl* mutant might be due to increased ethylene signaling.

To avoid the oxidative damage to other cells in plants, the ROS, such as superoxide anion (O2^−^) and H_2_O_2_, must be scavenged by the antioxidant enzymes SOD, CAT, APX, or GST etc. According to our results, O2^−^ and H_2_O_2_ are over-accumulated in leaves of *psl* mutant (Fig. 2). Therefore, up-regulation of antioxidant enzymes such as glutathione S-transferase might be a natural cellular response for scavenging excess ROS in *psl* mutant.

Increasing evidences suggest the function of the multifaceted regulator of GA signaling in plant stress response (De Vleesschauwer et al., 2016). The up-regulation of GID1L was also found during defense response to pathogen attack in plants (Zhang et al., 2015; Zhang et al., 2018). The induction of gibberellin receptor GID1L2 and calcium-dependent lipid-binding (CaLB) proteins were also identified in the *psl* mutant, supporting the function of GA signaling in plant stress and defense response.

### The *psl* mutant contain higher levels of ethylene

**Fig. 8:**
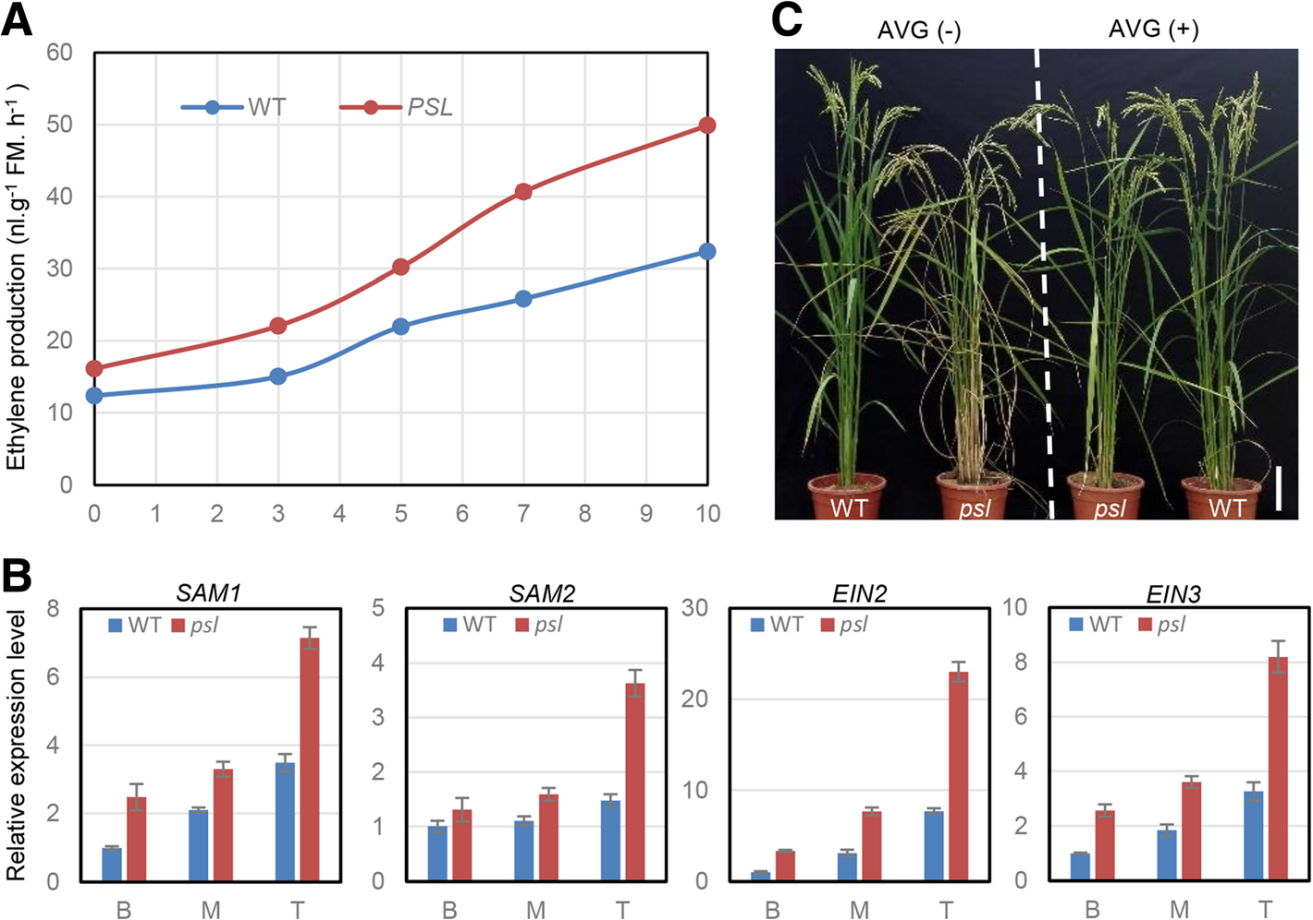
Ethylene is associated with leaf senescence in *psl.* **a** Ethylene content in WT and *psl*. **b** Expression of ethylene-synthesis and responsive genes were increased in the *psl* mutant. *25sRNA* was used as the internal control. **c** Application of exogenous AVG, an inhibitor of ethylene biosynthesis, retarded leaf senescence in *psl* mutant

As SAM synthesis is an early step in ethylene production in plants (Roje, 2006), it is possible that higher SAMS protein contents in *psl* mutants contributed to the ethylene synthesis. To test this possibility, we performed an ethylene assay. As expected, ethylene contents were higher in *psl* mutants than in WT plants (Fig. 8a). In addition, expression analysis showed that the transcript levels of ethylene-synthesis and responsive genes were increased in the *psl* mutant (Fig. 8b), consistent with the increased ethylene content. To ascertain whether the accumulation of ethylene was associated with the premature senescence phenotype of the *psl* mutant, the *psl* mutant and wild-type plants were treat with aminoethoxyvinylglycine (AVG), an ethylene biosynthesis inhibitor (Wang et al., 2002). Leaf senescence in *psl* mutants was relieved by the ethylene biosynthesis blocker (Fig. 8c). These results suggest that the accelerated senescence of *psl* plants may be associated with activation of the ethylene associated pathway.

## Discussion

In this study, we reported the identification and characterization of an early leaf senescence mutant (*psl*). The *OsPSL* gene was determined by a map-based cloning method. Its deduced amino acid sequence displays a high similarity to acetylglucosaminyl transferases family involved in protein *O*-glycosylation modification. During preparing for this article, Li et al. (Li et al., 2018) published premature leaf senescence mutant (*ospls3*). There are similarities between *psl* and *ospls3* mutants in many respects, including the increased level of ethylene. Therefore, it is not surprising that *psl* is an allele of *ospls3*. *ospls3* contains a 22-bp deletion, resulting in severe phenotypes, while the phenotypes are less severe in *psl* mutant, which have one amino acid deletion in conserved domain. Although several studies have uncovered functions for O-glycosylation in many key cellular processes (Zentella et al., 2016; Xu et al., 2017; Zentella et al., 2017), very few have been functionally related to senescence in plants. Taken with the findings by Li et al. (Li et al., 2018), it has been suggested that protein O-glycosylation modification might plays an important role in leaf senescence.

PCD is a fundamental process in plant development, environmental stresses and pathogens attacks. DNA fragmentation is a hallmark of PCD (Bröker et al., 2005). PBZ1 has been proposed as PCD marker protein in rice (Kim et al., 2008), which induced cell death possibly via its RNAse activity (Kim et al., 2011). In this study, we observed that the *psl* mutants display HR-like lesion in the absence of pathogens attacks (Fig. 1), ROS accumulation (Fig. 2) and clear DNA fragmentation (Fig. 3). Proteomic data showed that defense- or PCD-related-protein, including probenazole-induced protein (PBZ1), were significantly up-regulated in the *psl* mutant compared with WT. These results indicate that a mutation of *OsPSL* leads to PCD in rice.

Increasing evidences indicate that SAM synthetase (SAMS) is a broad-spectrum signaling molecule that regulates plant development and defense responses to various stresses through methylation reactions or ethylene biosynthesis pathway (Zhao et al., 2017). S-adenosylmethionine (SAM) is synthesized by SAM synthetase from methionine and ATP, and serves as a methyl group donor in secondary metabolism and a precursor of plant hormones ethylene. As methyl group, SAM is involved in methylation modification of DNA, RNA, proteins and lipids, and the synthesis of lignin monors, coniferyl and sinapyl alcohols, which require SAM-dependent methyltransferase (Roje, 2006). SAM metabolism is also required for ethylene production in plants. In this case, SAMS acts as a key component of the ethylene synthesis pathway and its activity is transcriptionally or post-translationally regulated in response to phytohormones, biotic and abiotic stress (Chen et al., 2013b; Mao et al., 2015; Jin et al., 2017). For example, *rice dwarf virus* (RDV) infection can enhance enzymatic activity of SAMS1 without upregulating its mRNA through an interaction of RDV-encoded protein Pns11 with OsSAMS1 and increase the production of ethylene (Zhao et al., 2017). Furthermore, it has been demonstrated that *SAMS* overexpression can confer plant hormone ethylene overproduction (Jin et al., 2017; Zhao et al., 2017). Therefore, the level of SAMS in plant cells is tightly associated with hormonal control of plant cell metabolism and development to adapt environmental and developmental signals. Consistent with the previous findings above mentioned, the expression of the *SAMSs* were substantially up-regulated in *psl* mutant (Fig. 8) and its protein levels were 6-fold higher than WT (Fig. 7), leading to higher ethylene level, which may contribute to the early senescence of *psl* mutant.

Protein *O*-glycosylation is a highly dynamic post-translational modification and plays an important role in regulating signal transduction pathways (Fülöp et al., 2008). *Arabidopsis* has two putative O-linked N-acetylglucosamine (O-GlcNAc) transferase (OGT): SPINDLY (SPY) and SECRET AGENT (SEC). Della, a master growth repressors in plants by inhibiting phytohormone gibberellin (GA) signaling, have been shown to be subject to O-GlcNAc modification by O-linked N-acetylglucosamine (O-GlcNAc) transferase (OGT) SECRET AGENT (SEC) in *Arabidopsis* (Zentella et al., 2016). Recently, Zentella, et al. reported that SPY is a protein O-fucosyltransferase and SPY and SEC competed with each other in modifying Della protein RGA (Zentella et al., 2017). Rice has three putative OGTs genes: SPY, SEC1 and SEC2 (Shimada et al., 2006). Genetic evidence indicates that OGTs is as important in rice as in *Arabidopsis* (Shimada et al., 2006). Interestingly, the expression levels of rice *OGTs* were severely down-regulated in *psl* mutants compare to WT, suggesting the possible alteration of O-glycosylation modification, including rice DELLA protein SLR1. Thus, this might have an effect on GA signaling pathway. In support of this, the expression of *OsGID1*, encoding gibberellin receptor protein, was significantly suppressed in *psl* mutant. In addition, our data shows that defense-related proteins such as PBZ1/OsPR10 are over accumulated in *psl* mutant (Fig. 7), as observed in a rice GA-insensitive dwarf mutant *gid1*(Tanaka et al., 2005). These results support the putative role of OsPSL in O-glycosylation protein modification. It is therefore possible that the early leaf senescence of *psl* mutant may be associated with suppression of GA pathway and activation of ethylene-associated pathway, due to impaired O-glycosylation protein modification in leaf tissue of *psl* mutant. Our study reveals the potential role of OsPSL in regulating leaf senescence. However, the biochemical function of OsPSL remains unsolved. Future studies should uncover the biochemical function of OsPSL and identify its target proteins.

## Conclusions

In the present study, we propose that OsPSL plays an important role in regulating PCD and leaf senescence in rice. In addition, our results suggest the early leaf senescence of *psl* mutant may be associated with activation of ethylene-associated pathway, due to impaired protein glycosylation.

## Additional files

Additional file 1: **Figure S1.** Comparison of nucleotide sequences between WT and *psl* mutant. **Figure S2.** Three-dimensional structure prediction of OsPSL protein. **Figure S3.** Representative 2-D gel protein profiles of leaves from WT and *psl* mutant. **Table S1.** List of primers used in this study. **Table S2.** Comparisons of agronomic traits between WT and *psl* mutant. (PDF 477 kb)

## Abbreviations

2-DGE: Two-dimensional gel electrophoresis
6PGDH: 6-phosphogluconate dehydrogenase
AVG: Aminoethoxyvinylglycine
C2GnT: Core 2/I branching beta-1,6-N-acetylglucosaminyl transferases
DAB: 3,3^′^-diaminobenzidine
GalNAc: N-acetylgalactosamine
GAPDH: Glyceraldehyde-3-phosphate dehydrogenase
lmm: Lesion mimic mutants
NBT: Nitro blue tetrazolium
OGTs: O-linked N-acetylglucosamine (O-GlcNAc) transferases
PBZ1: Probenazole-induced protein
PCD: Programmed cell death
ROS: Reactive oxygen species
SAMS: S-adenosyl methionine (SAM) synthetase
