# Supplementary material for: Mutation in a putative glycosyltransferase-like gene causes programmed cell death and early leaf senescence in rice
Source: Rice (N Y). 2019 Feb 13;12:7. doi: 10.1186/s12284-019-0266-1 (PMC6374497; doi:10.1186/s12284-019-0266-1)
Supplement: Supplementary file 1 — Figure S1. Comparison of nucleotide sequences between WT and psl mutant. Figure S2. Three-dimensional structure prediction of OsPSL protein. Figure S3. Representative 2-D gel protein profiles of leaves from WT and psl mutant. Table S1. List of primers used in this study. Table S2. Comparisons of agronomic traits between WT and psl mutant. (PDF 477 kb) [file 12284_2019_266_MOESM1_ESM.pdf]

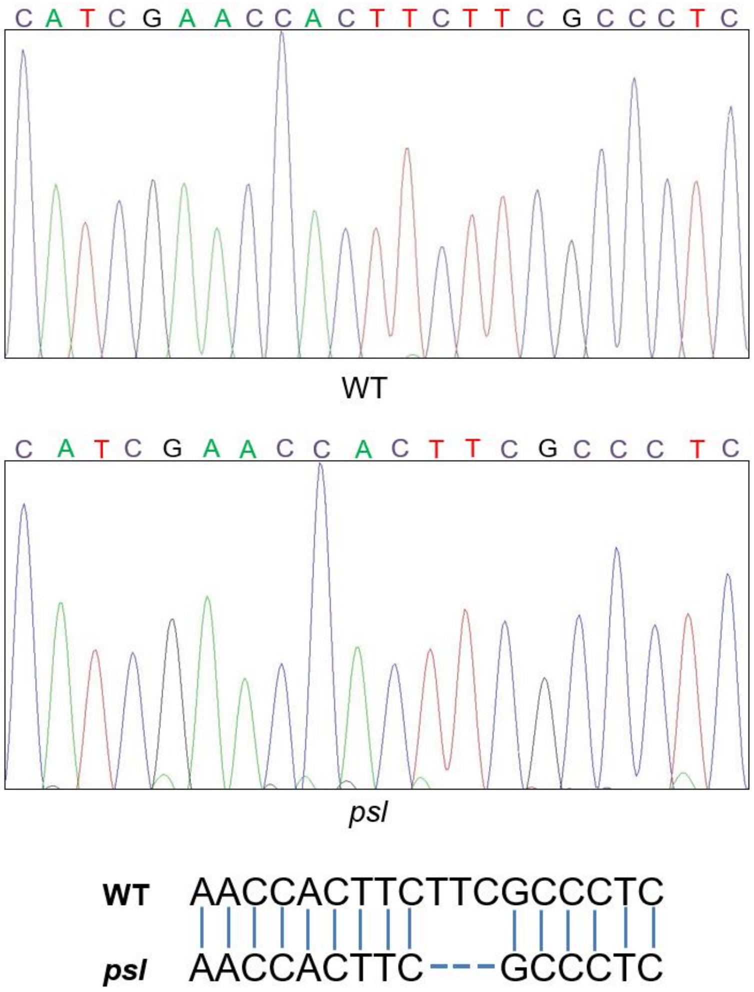

Figure S1. Comparison of nucleotide sequences between WT and *psl* mutant. Nucleotide sequence alignment revealed that there was a deletion of 3 bp in the exon of LOC_Os12g42420 in the *psl*.

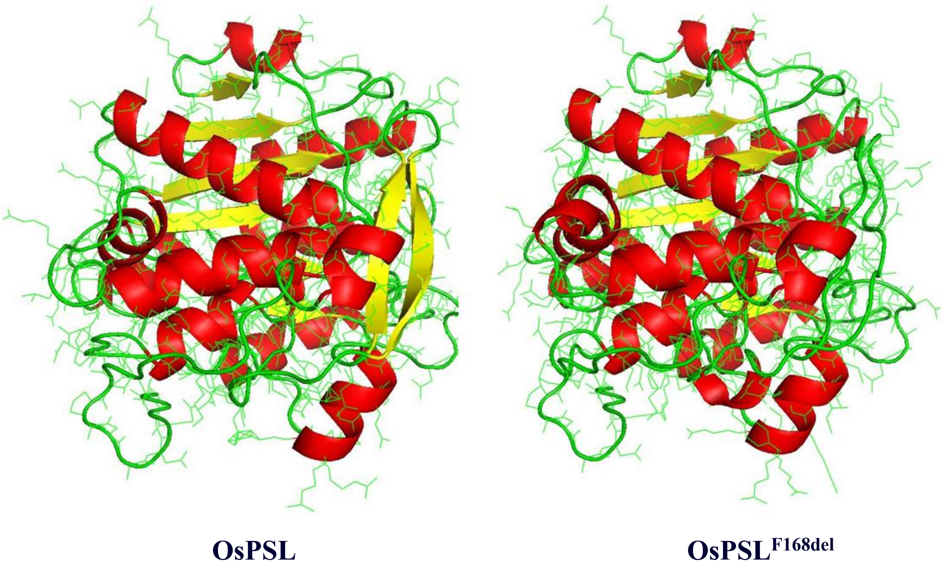

Figure S2. Three-dimensional structure prediction of OsPSL protein. The advanced structure of the OsPSL protein was predicted by using Phyre2 server (http://www.sbg.bio.ic.ac.uk/phyre2). A phenylalanine residue (F168) is located in a *β*-sheet and its deletion leads to the absence of the *β*-sheet in OsPSL protein

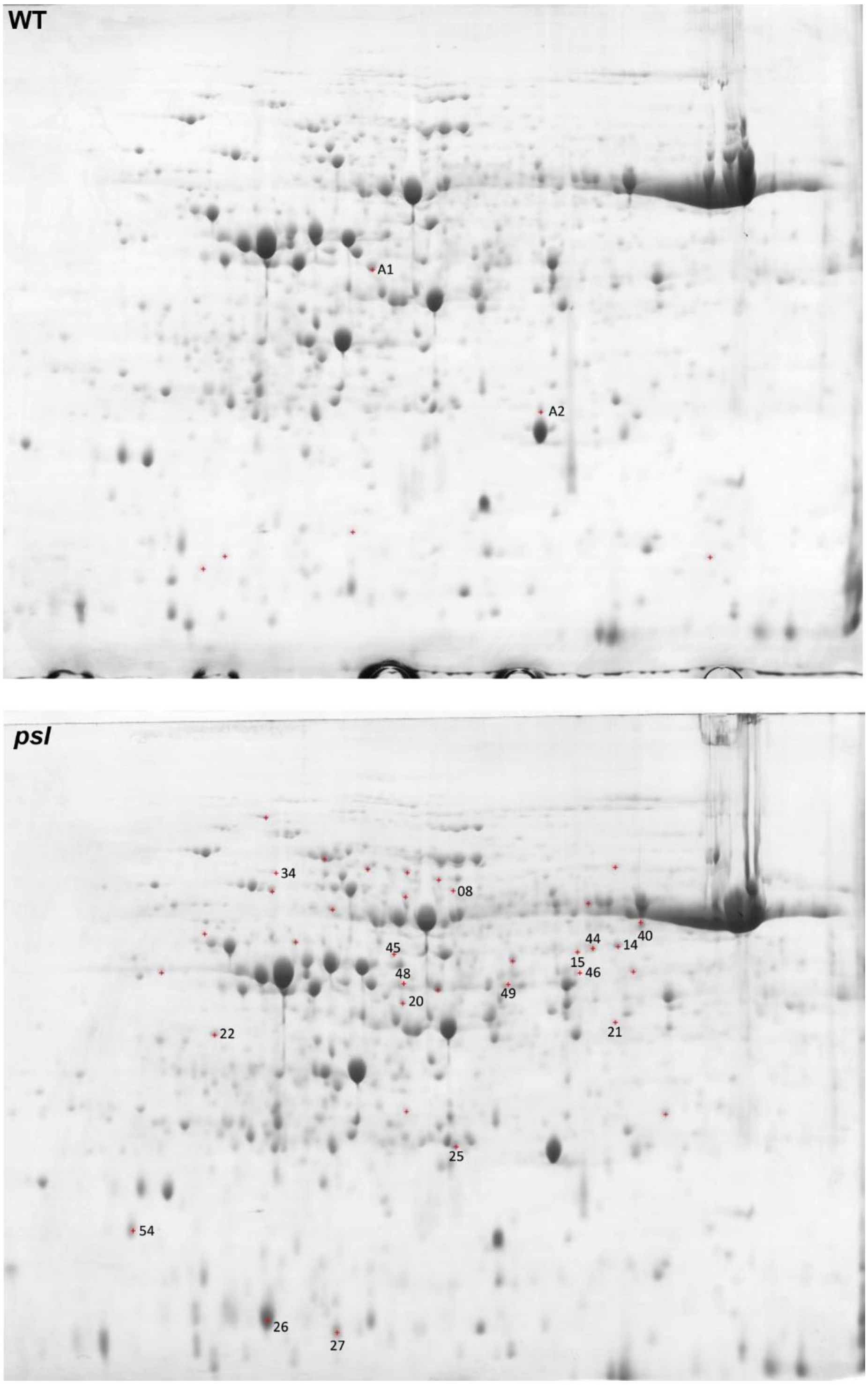

Figure S3. Representative 2-D gel protein profiles of leaves from the *psl* mutant and WT. Coomassie blue stained spots were detected by ImageMaster software in the WT and *psl* gel profiles, respectively. Numbered “+” show proteins selected for mass spectrometry.

Table S1 List of primers used in this study.

Primers	Sequence (5′→ 3′)	Locus/Gene	Experiments	Marker's type
PSM183F	GACAAAACCGGCAACAGATT	Chromosome 12	Map-based cloning	SSR
PSM183R	TAGCTTTATTGTGGCAGGGG	Chromosome 12	Map-based cloning	
RM247F	TAGTGCCGATCGATGTAACG	Chromosome 12	Map-based cloning	SSR
RM247R	CATATGGTTTTGACAAAGCG	Chromosome 12	Map-based cloning	
PSM419F	GAGGCAATAGGAGGGGAGA	Chromosome 12	Map-based cloning	SSR
PSM419R	GGCAGCGAAAGCATAAAGTA	Chromosome 12	Map-based cloning	
PSM420F	ACCTATGGCTTGCGACGAAC	Chromosome 12	Map-based cloning	SSR
PSM420R	CTGAGTTGACAATATCATCACGGA	Chromosome 12	Map-based cloning	
PSM421F	TTGGCGTGCTGCTGTATTTT	Chromosome 12	Map-based cloning	SSR
PSM421R	GTCCCGGATTCATGGTTCTG	Chromosome 12	Map-based cloning	
RM519F	AGAGAGCCCCTAAATTTCCG	Chromosome 12	Map-based cloning	SSR
RM519R	AGGTACGCTCACCTGTGGAC	Chromosome 12	Map-based cloning	
PSM464F	CCGTTCAAATGTTAATGTCCAG	Chromosome 12	Map-based cloning	SSR
PSM464R	GACCAAAGGGTGAGACCAATC	Chromosome 12	Map-based cloning	
PSM189F	TGATAGTTGGACCCGATCCT	Chromosome 12	Map-based cloning	SSR
PSM189R	ATCACCCCTCTGCTCGTCT	Chromosome 12	Map-based cloning	
RM235F	AGAAGCTAGGGCTAACGAAC	Chromosome 12	Map-based cloning	SSR
RM235R	TCACCTGGTCAGCCTCTTTC	Chromosome 12	Map-based cloning	
PSM192F	CTGCAGCCCTGTGATAGGAT	Chromosome 12	Map-based cloning	SSR
PSM192R	TGTGCGGTTGCTTTCATAGA	Chromosome 12	Map-based cloning	
PSM193F	CATCGTCCTCGGTATCATCA	Chromosome 12	Map-based cloning	SSR
PSM193R	GTGAAACGGAGGGAGTAGCA	Chromosome 12	Map-based cloning	
AL732532-2F	AACCCACGGGTTTGACTAGC	Chromosome 12	Map-based cloning	InDel
AL732532-2R	GTCTGGTAGCTGGGATCGTG	Chromosome 12	Map-based cloning	
BX000457-1F	CGGATATACACGGCTGCACT	Chromosome 12	Map-based cloning	InDel
BX000457-1R	TGTTAGAAGGGTGCTGAGGC	Chromosome 12	Map-based cloning	
AL731885-1F	CTGTTTGCTCGTTCGTTGGA	Chromosome 12	Map-based cloning	InDel
AL731885-1R	TCGACCCAACCATCACCTG	Chromosome 12	Map-based cloning	
AL731885-3F	AAGTGACAGGAGTGGCATGG	Chromosome 12	Map-based cloning	InDel
AL731885-3R	GGATAGCTTGGTTCGTCCGT	Chromosome 12	Map-based cloning	
AL845347-11F	GGTGGCCCATCATGTCAGAG	Chromosome 12	Map-based cloning	InDel
AL845347-11R	GCAATGCTAACACCTCGCAT	Chromosome 12	Map-based cloning	
AL845347-13F	TGCTAAGGGAGTCAGAACGC	Chromosome 12	Map-based cloning	InDel
AL845347-13R	AGATCGCGAACACGCAAAAG	Chromosome 12	Map-based cloning	
AL732532-7F	TGTGTGCCTTACAGCAGAGG	Chromosome 12	Map-based cloning	InDel
AL732532-7R	TCCCGCTAGAGAGGGTCAC	Chromosome 12	Map-based cloning	
AL732532-8F	ATTTTGCGTCGAATTGGGGC	Chromosome 12	Map-based cloning	InDel
AL732532-8R	TCGCACAAACCCTAGAACCG	Chromosome 12	Map-based cloning	
AL732532-5F	AGCGTGAACTAAACACACCCA	Chromosome 12	Map-based cloning	InDel
AL732532-5R	TCTCCTCCAGAATCGCTCCA	Chromosome 12	Map-based cloning	

Table S1 List of primers used in this study (continued).

Primers	Sequence (5′→ 3′)	Locus/Gene	Experiments
PSLF	GGTACCTCTAGAATGGCGCTGCCGCACGC CGCCT	LOC_Os12g42420	Complement assay
PSLR	ACTAGTCTACCATGGGTCTCGCAGGATGA CGGA		Complement assay
PSL-qRTF	CACCCTTTCCCCACCTTCTACC	LOC_Os12g42420	qRT-PCR
PSL-qRTR	GAACTTGTTCCACAGCCTCCGA		qRT-PCR
SEC1-F	GAGCACATCAGACGCAGTGC	LOC_Os01g68680	qRT-PCR
SEC1-R	CCATCTCTTCCCCAATTCCA		qRT-PCR
SEC2-F	TTCCTGGACACCCCTCTCTG	LOC_Os02g28830	qRT-PCR
SEC2-R	AATCATCTCTTCCCCTAGCCC		qRT-PCR
SPY-F	GAGGCTGGTTGCGAAGTCTG	LOC_Os08g44510	qRT-PCR
SPY-R	TGCGCCACATGTTTCTGTATG		qRT-PCR
EIN2F	TACTGCCTAACTGCGGAGACG	LOC_Os07g06130	qRT-PCR
EIN2R	CCAGGATGCCCTGAAGACG		qRT-PCR
EIN3F	CACGGAGAACAAGCCATCG	LOC_Os03g20790	qRT-PCR
EIN3R	GCCACTTCCCTGTCCAAAAA		qRT-PCR
B14F	ATCAAGCCTGTCATTCCCGA	LOC_Os05g04510	qRT-PCR
B14R	GAGCTCCCCAGCCACCATA		qRT-PCR
B15F	CCATCTTCCACCTCAACCCC	LOC_Os01g22010	qRT-PCR
B15R	CTGGCGACGATGCTCTTGG		qRT-PCR
25SR-F	AAGGCCGAAGAGGAGAAAGGT	25sRNA	qRT-PCR
25SR-R	TTGGCGGGCCGTTAAGCAGAAAAGA		

Table S2 Comparisons of agronomic traits between WT and *psl* mutant.

Trait	WT	*psl*
Heading date (d)	81.33 ± 0.58	81.67 ± 1.02
Seed setting rate (%)	84.47 ± 3.16	79.56 ± 2.58*
1000-grain weight (g)	26.52 ± 0.16	23.51 ± 0.27**
Plant height (cm)	86.16 ± 2.02	86.07 ± 1.76

Asterisks represent significant difference at p-value<0.05(*) or <0.01(**).
